# Modifying angular and polarization selection rules of high-order harmonics by controlling electron trajectories in k-space

**DOI:** 10.1038/s41467-020-16875-5

**Published:** 2020-06-17

**Authors:** Yasuyuki Sanari, Tomohito Otobe, Yoshihiko Kanemitsu, Hideki Hirori

**Affiliations:** 1 Institute for Chemical Research, Kyoto University, Uji, Kyoto 611-0011 Japan; 2 Kansai Photon Science Institute, National Institutes for Quantum and Radiological Science and Technology (QST), Kizugawa, Kyoto 619-0215 Japan

**Keywords:** High-harmonic generation, Nonlinear optics, Ultrafast photonics

## Abstract

Recent advances in generation of strong laser pulses have enabled the acceleration of electrons in solids into regions far away from the band edge. Because nonlinear currents can be generated by laser-driven carriers in the non-parabolic region, tailored laser fields may allow control of optical properties of high-order harmonics (HHs). So far, investigations on laser-induced nonlinear optical phenomena have focused on the simple electron motion induced by linearly or elliptically polarized fields. However, more complex trajectories can be important for development of novel optoelectronic devices. Here, we show that a weak laser field (optical frequency is *ω*_2_) applied in a direction orthogonal to a strong main field (*ω*_1_) enhances certain HH intensity components of bulk GaSe by a factor of 100. Good agreement between the experiments and calculations shows that manipulation of the electron trajectory allows breaking inversion symmetry of the electronic states felt by the accelerated electrons and leading to a modification of selection rules for frequency-mixing processes of HHs. Owing to our usage of non-integer multiples for *ω*_1_ and *ω*_2_, it is found that the generation of HHs constitutes a novel way of ultrafast control of light polarization and optical switching.

## Abstract (web summary)

High harmonic generation from solids is an interesting and growing field of research. Here the authors explore HHG from GaSe sample driven by two-color fields and find that controlling electron trajectories in a band structure enable to modify the angular and polarization selection rules of HHG.

## Introduction

Soon after the first demonstration of the laser, experiments on nonlinear optics received significant attention, and this led to the development of numerous nonlinear optics technologies^1,2^. Recently, a nonlinear optical effect called high-order harmonic generation (HHG) has been found to occur in solids upon irradiation with an intense laser pulse^3–10^. This discovery has opened the door to a new era in nonlinear optics, as this effect enables generation of pulses in the extreme ultraviolet wavelength region. In order to implement HHG in devices, research on the fundamentals of HHG in solids is ongoing; it has been proposed that the mechanism of HHG in solids is different from that in atoms and molecules^11,12^. In particular, the nonlinear intraband current generated by acceleration of carriers in certain bands in k-space as well as coherent interband transitions are considered to be important sources of high-order harmonics (HHs). For this reason, experiments on HHG induced by specific laser polarization states have been conducted with the aim of understanding the underlying generation mechanism and controlling the efficiency of HHG by manipulating the motion of carriers^13–16^.

One way of manipulating the carrier motion in a band is to use the electric fields of two orthogonally polarized lasers at frequencies *ω*_1_ and *ω*_2_^17–19^. The HHs arising from carriers driven by two lasers include more information than those generated by carriers driven in one dimension, and such information will be advantageous for band reconstruction methodologies^20^. In addition, the mixing processes in conjunction with HHG are essential to frequency-mixing technologies such as sum/difference frequency generation and optical frequency combs for fine optical waveform synthesis^21–24^. Besides these applications, manipulation of electron trajectories by multiple laser fields should allow us to control the symmetry of the electronic states felt by the electrons. In this way, we can utilize potentials that are more complex than the band structure topology accessible by single fields, and may discover novel aspects regarding the control of HH emission on the ultrafast time scale. Recently, there are studies that used two orthogonally polarized fields where *ω*_2_ is an integer multiple of *ω*_1_, such as the elliptic case *ω*_1_ = *ω*_2_ = *ω*^13^. In general, the observed *a*th-order harmonic peak includes all components labeled with integers (*b*,*c*) that satisfy the relation *aħω* = *bħω*_1_ + *cħω*_2_, and these components cannot be separated experimentally. For this reason, it is not possible to distinguish the origin of each (*b*,*c*) component, and thus this excitation scheme is still not enough to discuss the relationship between the motion of carriers and HHG.

Here, we study the HHG in bulk GaSe that occurs upon excitation by two orthogonally polarized laser fields **E**_1_(*t*) and **E**_2_(*t*) with amplitudes *E*_1_ and *E*_2_ and different optical frequencies (*ω*_1_ and *ω*_2_). The photon energy of *ħω*_1_ = 0.517 eV (*λ*_1_ = 2.4 μm) and *ħω*_2_ = 0.954 eV (*λ*_2_ = 1.3 μm) was used in the experiment, where *ω*_2_ is non-integer multiples for *ω*_1_ (*ω*_2_ = 1.85*ω*_1_). The peaks of the generated spectra occur at photon energies equal to *mħω*_1_ + *nħω*_2_, where *m* and *n* are integers that denote the harmonic order. We investigate the crystal-angle dependences of the HH intensity components along the two orthogonal directions, and find a behavior that cannot be explained by the model developed for the case of linearly polarized excitation. The results are explained with a theoretical model, showing the importance of a two-dimensional (2D) electron trajectory for HH polarization control.

## Results

**Fig. 1:**
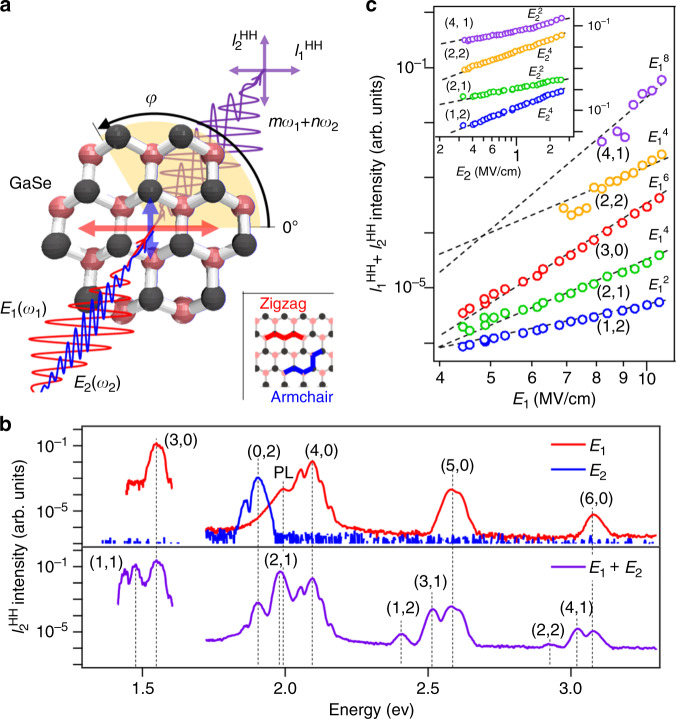
HHG with two orthogonally polarized optical fields. **a** Experimental setup. Two orthogonally polarized pulses with photon energies *ħω*_1_ and *ħω*_2_ and field strengths *E*_1_ and *E*_2_ are focused onto the sample (bulk GaSe with a thickness of 30 μm). The generated HHs (observed at the energies *mħω*_1_ + *nħω*_2_) are decomposed along the two mutually orthogonal directions of the electric fields **E**_1_(*t*) and **E**_2_(*t*), and their intensities, $I_1^{{\mathrm{HH}}}$ and $I_2^{{\mathrm{HH}}}$, are measured as a function of the crystal rotation angle *φ* (rotation axis: pulse propagation direction). **b** The $I_2^{{\mathrm{HH}}}$ component in the HH spectra separately obtained by excitation with **E**_1_(*t*) *(*red data), **E**_2_(*t*) (blue data), and **E**_1_(*t*) + **E**_2_(*t*) (purple data) for *φ* = 15°. To distinguish the different mixed harmonic orders, we use ordered pairs, (*m*,*n*). Photoluminescence (PL) appears around 2 eV^31^. **c** Harmonic intensities as a function of *E*_1_ for the harmonic orders (*m*,*n*) = (4,1), (2,2), (3,0), (2,1), (1,2) (solid circles), while *E*_2_ = 0.5 MV/cm. The black broken lines are proportional to *E*^2*m*^ and serve as guides for the eye. The data sets are offset for clarity. Inset: *E*_2_ dependences of the (4,1)-, (2,2)-, (1,2)- and (2,1)-order harmonics’ intensities, while *E*_1_ = 5 MV/cm; the black broken lines are proportional to *E*^2*n*^.

Figure 1a shows the experimental setup. The experimental details are provided in “Methods” section. We use the convention that the rotation angle *φ* of the GaSe sample is 0° when the polarization direction of **E**_1_(*t*) is parallel to the zigzag direction of GaSe. The electric-field components of the HHs along the directions of **E**_1_(*t*) and **E**_2_(*t*) are denoted by $E_1^{{\mathrm{HH}}}$ and $E_2^{{\mathrm{HH}}}$, and their corresponding intensities are $I_1^{{\mathrm{HH}}}$ and $I_2^{{\mathrm{HH}}}$. The red and blue data in the upper panel of Fig. 1b plot the $I_2^{{\mathrm{HH}}}$ components of the HH spectra obtained under excitation with **E**_1_(*t*) only (*E*_1_ = 10 MV/cm, *E*_2_ = 0 MV/cm) and with **E**_2_(*t*) only (*E*_1_ = 0 MV/cm, *E*_2_ = 0.5 MV/cm), respectively. Here, *φ* = 15° and HHG up to the sixth order for **E**_1_(*t*) and up to the second order for **E**_2_(*t*) can be confirmed. The lower panel shows the $I_2^{{\mathrm{HH}}}$ data obtained when applying **E**_1_(*t*) + **E**_2_(*t*) simultaneously. Under this excitation condition, peaks appear at photon energies of *mħω*_1_ + *nħω*_2_. The use of incommensurate frequencies for **E**_1_(*t*) and **E**_2_(*t*) (i.e., *mω*_1_ ≠ *nω*_2_ for any positive integer *m, n*) allows us to avoid overlap of HHs with different orders and to clarify the angle dependence of each order [the HH orders are labeled by ordered pairs (*m*,*n*)]. The data indicate that the HH spectrum spreads by simply adding a weak electric-field pulse to the excitation. Figure 1c shows the dependences of several harmonic orders on the field strength *E*_1_ for *φ* = 15°. Here, each data line plots the integrated HH intensity $I_1^{{\mathrm{HH}}} + I_2^{{\mathrm{HH}}}$, which is proportional to the 2*m*th power of *E*_1_ (shown with the black broken curves). The inset of Fig. 1c clarifies that the dependence of the integrated HH intensity on *E*_2_ also follows such a law, i.e., $E_2^{2n}$.

**Fig. 2:**
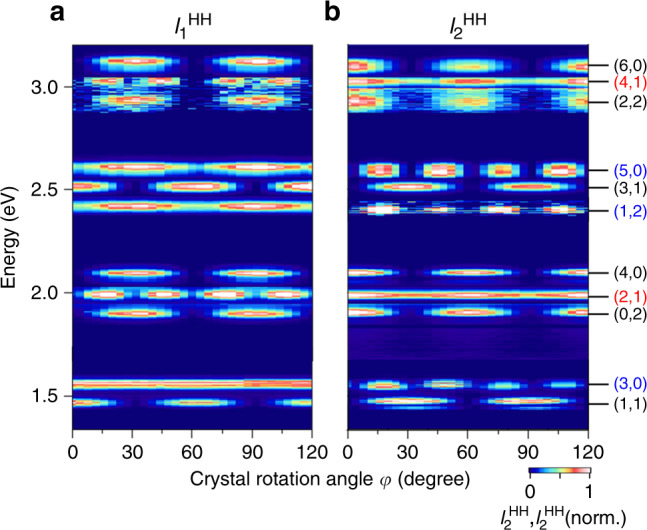
Crystal-angle dependence of high-order harmonics. **a** Dependence of $I_1^{{\mathrm{HH}}}$ on the crystal rotation angle *φ* for *E*_1_ = 10 MV/cm and *E*_2_ = 0.5 MV/cm. The generated orders of the harmonics at energies *mħω*_1_ + *nħω*_2_ are labeled by (*m*,*n*). **b**, Dependence of $I_2^{{\mathrm{HH}}}$ on *φ*.

**Fig. 3:**
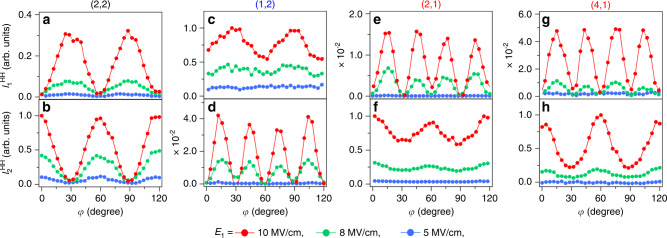
Crystal-angle dependence as a function of the field strength. **a** Dependence of the $I_1^{{\mathrm{HH}}}$ component on *φ* for the (2,2) order as a representative of the orders with *m* + *n* = even for different values of *E*_1_ (5, 8, and 10 MV/cm) and *E*_2_ = 0.5 MV/cm. **b** Dependence of $I_2^{{\mathrm{HH}}}$ on *φ* and *E*_1_. **c**–**h** Corresponding dependences for the (2,2), (1,2), (2,1), and (4,1) orders. (Either $I_1^{{\mathrm{HH}}}$ or $I_2^{{\mathrm{HH}}}$ for each order is normalized, and their intensity ratios for the same order are maintained). These orders are representative of the HH orders with *m* + *n* = odd, where *m* is either odd or even.

The 2D maps for the crystal rotation angle (*φ*) dependences of the $I_1^{{\mathrm{HH}}}$ and $I_2^{{\mathrm{HH}}}$ components are provided in Fig. 2a, b, respectively. These maps exhibit a sixfold rotation symmetry for harmonic orders (*m*,*n*) that fulfill the restriction *m* + *n* = even, which are indicated by the black labels on the right side of the figure. Figure 3a, b shows the *φ* dependences of $I_1^{{\mathrm{HH}}}$ and $I_2^{{\mathrm{HH}}}$ for the (2,2) order, which is representative of orders with *m* + *n* = even. We note that $I_1^{{\mathrm{HH}}}$ vanishes at *φ* = 0, 60 and 120° (Fig. 3a), while $I_2^{{\mathrm{HH}}}$ vanishes at *φ* = 30, and 90° (Fig. 3b). These even orders are assigned to the HH components caused by interband polarization, and the sixfold rotation symmetry of the harmonic orders obeying *m* + *n* = even can be explained by conventional nonlinear polarization^25–27^ (Supplementary Fig. 1a–d in Supplementary Information I). Also the behaviors of components with *m* + *n* = odd observed under weak excitation conditions can be explained by perturbative nonlinear optics (Supplementary Fig. 1e, f).

On the other hand, the components of HH signals that contain both even and odd orders (i.e., *m* + *n* = odd) exhibit either six- or twelve-fold rotation symmetries. For example, in the case of *m* = even, i.e., (2,1) and (4,1), as indicated by the red labels in Fig. 2, a 12-fold rotation symmetry appears for $I_1^{{\mathrm{HH}}}$, while in the case of *m* = odd, i.e., (3,0), (1,2), and (5,0) indicated by the blue labels, the 12-fold rotation symmetry appears for $I_2^{{\mathrm{HH}}}$^28,29^. Despite the fact that the HH intensities obey power laws (Fig. 1c), Fig. 3c–h evidence that the *φ* dependences of $I_1^{{\mathrm{HH}}}$ and $I_2^{{\mathrm{HH}}}$ are modified when *E*_1_ is changed, suggesting the onset of a non-perturbative interaction. (Further stronger electric-field dependences are shown in Supplementary Fig. 2 of the Supplementary Information II.)

Figure 3c, d clarify the *φ* dependences of $I_1^{{\mathrm{HH}}}$ and $I_2^{{\mathrm{HH}}}$ for the (1,2) order as a representative for the group of (odd and even) orders. When *E*_1_ is weak (5 MV/cm), $I_1^{{\mathrm{HH}}}$ is almost constant and $I_2^{{\mathrm{HH}}}$ is zero. As *E*_1_ increases, six- and twelve-fold rotation symmetries appear in the *φ* dependences of $I_1^{{\mathrm{HH}}}$ (Fig. 3c) and $I_2^{{\mathrm{HH}}}$ (Fig. 3d), respectively. Note that the HH intensity along **E**_2_(*t*) is much weaker than that along **E**_1_(*t*), which may be considered trivial since *E*_2_ < *E*_1_. For interpretation of this behavior, we consider that the electrons mainly move in one dimension, because *E*_2_ is sufficiently weak. Consequently, the electron is driven almost along the same trajectory as in the case of excitation by **E**_1_(*t*) only. This suggests that the electrons accelerated in the direction of **E**_1_(*t*) will induce the nonlinear current resembling that by single laser field^29^ and that the six- and twelve-fold rotation symmetries appear parallel and perpendicular to the excitation field, respectively.

Nonetheless, for the harmonic orders (2,1) and (4,1), which are representative of the group of (even and odd) orders, twelve- and six-fold rotation symmetries appear in the *φ* dependences of $I_1^{{\mathrm{HH}}}$ (Fig. 3e, g) and $I_2^{{\mathrm{HH}}}$ (Fig. 3f, h), respectively. Despite the nearly one-dimensional motion, strong HH intensity components $I_2^{{\mathrm{HH}}}$ are observed along the direction orthogonal to the main field **E**_1_(*t*). Actually, these components of the HHs that are orthogonal to the main optical field polarization are two order of magnitudes larger than the HH intensities parallel to **E**_1_(*t*).

The peculiar behavior of $I_2^{{\mathrm{HH}}}$ shown Fig. 3f, h indicates that the much weaker but finite optical field **E**_2_(*t*) causes a non-negligible modification of the electron motion, and allows generation of a strong nonlinear current and HH emission. A model that considers only a single linearly polarized excitation cannot describe these features, since such a model reduces to a description employing a uniquely defined band curvature for any given pair of polarization angle *φ* and absolute wave vector |**k**|(except at |**k**| = 0). However, in general, a 2D electron trajectory **k**(*t*) established for a certain *φ* can cross another trajectory that is established for a different polarization angle, and therefore the nonlinear current (proportional to the band curvature felt by the driven electron) is not uniquely defined for a given *φ* and |**k**|. In order to describe the *φ* dependences of the (even and odd) orders, we need a physical model that takes into account the 2D motion of electrons in **k**-space.

To calculate the electric-field waveform of HHs, we consider that the two orthogonally polarized fields, **E**_1_(*t*) = *E*_1_ cos(*ω*_1_*t*)**e**_1_ and **E**_2_(*t*) = *E*_2_ cos(*ω*_2_*t*)**e**_2_ where **e**_1_ and **e**_2_ denote two orthogonal unit vectors, accelerate the electron two-dimensionally in the band, thereby inducing an intraband current **J**_intra_(*t*)^29,30^ (Supplementary Information III). The two mutually orthogonal components *E*_*i*_ (*i* = 1,2) of the HHs can be related to the electron wave packet **k**(*t*) in **k**-space, the **J**_intra_(*t*) = (*j*_1_(*t*),*j*_2_(*t*)), and the band dispersion *ε*(**k**) as follows:

1 $$\left( {\begin{array}{*{20}{c}} {E_1^{{\mathrm{HH}}}\left( t \right)} \\ {E_2^{{\mathrm{HH}}}\left( t \right)} \end{array}} \right) 	 \propto \frac{d}{{dt}}\left( {\begin{array}{*{20}{c}} {J_1\left( t \right)} \\ {J_2\left( t \right)} \end{array}} \right) = - \frac{e}{\hbar }\frac{d}{{dt}}\left( {\begin{array}{*{20}{c}} {\frac{{d{\it{\epsilon }}\left( {{\mathbf{k}}\left( t \right)} \right)}}{{dk_1}}} \\ {\frac{{d{\it{\epsilon }}\left( {{\mathbf{k}}\left( t \right)} \right)}}{{dk_2}}} \end{array}} \right)\\ 	= - \frac{e}{\hbar }\left( {\begin{array}{*{20}{c}} {\frac{{\partial ^2{\it{\epsilon }}\left( {\mathbf{k}} \right)}}{{\partial k_1^2}}} & {\frac{{\partial ^2{\it{\epsilon }}\left( {\mathbf{k}} \right)}}{{\partial k_1\partial k_2}}} \\ {\frac{{\partial ^2{\it{\epsilon }}\left( {\mathbf{k}} \right)}}{{\partial k_2\partial k_1}}} & {\frac{{\partial ^2{\it{\epsilon }}\left( {\mathbf{k}} \right)}}{{\partial k_2^2}}} \end{array}} \right)\left( {\begin{array}{*{20}{c}} {E_1{\mathrm{cos}}\left( {\omega _1t} \right)} \\ {E_2{\mathrm{cos}}\left( {\omega _2t} \right)} \end{array}} \right).$$

Here, *e* is the elementary charge and ${\hbar}$ is the Dirac constant. Equation (1) reveals that the HHs originating from the electron motion in the band reflect a 2 × 2 matrix containing elements for the band curvatures (inverse effective masses) that describe how the band changes along the field directions. Note that the intraband current shown in Eq. (1) depends solely on the first derivative (or curvature) of the band. Furthermore, since **E**_1_(*t*) and **E**_2_(*t*) are orthogonal, all four matrix elements are reflected in the HHs.

**Fig. 4:**
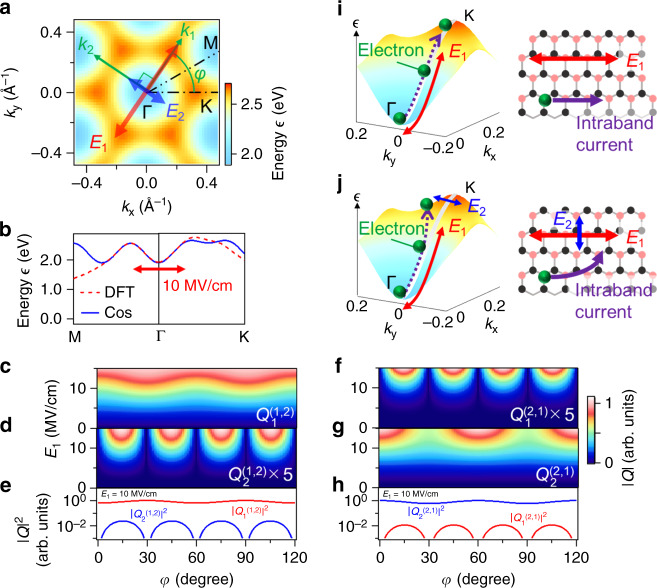
Calculation model that considers 2D electron motion and the theoretical results. **a** 2D band dispersion of the first conduction band modeled by employing a cosine function. **b** Comparison of the band structures obtained by density-functional theory (DFT) and the model that uses a cosine band structure. The red arrow indicates the region where the electric field with *E*_1_ = 10 MV/cm is able to drive the electrons. **c**–**h** *Q*_*i*_^(1,2)^ and *Q*_*i*_^(2,1)^ (*i* = 1,2) as functions of *E*_1_ and *φ* calculated by using Eq. (2). **i** Schematic of the motion of electrons driven by a single linearly polarized laser field in **k**-space and real space. **j** Schematic for excitation with two orthogonal laser fields.

Figure 4a plots the 2D band profile used in our calculation, where the band dispersion of the first conduction band was modeled by assuming a cosine band structure. The band structures obtained by density-functional theory (DFT) and the model based on the cosine band structure are compared in Fig. 4b. The maximum wavenumber that can be achieved with *E*_1_ = 10 MV/cm is $eE_1/\hbar \omega _1$ = 0.19 Å^−1^, which is indicated by the red arrow, and in this range of wavenumbers, both models show good agreement. Thus, the approximation using the cosine band structure is sufficiently accurate to explain the experimental results. By expanding the result in a series of Bessel functions of the first kind^6^, the four elements in Eq. (1) for the band curvatures can be obtained. The resulting analytical formula for the HHs consists of oscillation terms of type cos[(*mω*_1_ + *nω*_2_)*t*] summed over non-negative integers *M*, *N*:

2 $$E_{i}^{{\mathrm{HH}}} \propto \mathop {\sum}\limits_{M,N} \left\{ Q_{i}^{\left( {2M, \pm (2N + 1)} \right)}\left( {E_1,E_2,\varphi } \right)\cos \left[ {2M\omega _1t \pm \left( {2N + 1} \right)\omega _2t} \right]\right.\\ + \left.\;Q_i^{\left( {2M + 1, \pm 2N} \right)}\left( {E_1,E_2,\varphi } \right)\cos \left[ {\left( {2M + 1} \right)\omega _1t \pm 2N\omega _2t} \right] \right\}\;\left( {i = 1,2} \right)$$

The coefficient $Q_i^{\left( {m,n} \right)}$ determines the *φ* dependence of the electric-field amplitude of the harmonic order (*m*,*n*) (Supplementary Information III).

To explain the experimentally observed *φ* dependences in Fig. 3c–f, we show the calculated $Q_i^{\left( {1,2} \right)}$ and $Q_i^{\left( {2,1} \right)}$ as functions of *E*_1_ and *φ* in Fig. 4c–f (*E*_2_ is fixed at 0.5 MV/cm). In the region of weak *E*_1_ (below 5 MV/cm), the predicted efficiencies do not significantly depend on the crystal orientation. Nonetheless, the six- (twelve-) fold rotation symmetry for $Q_1^{\left( {1,2} \right)}$ ($Q_2^{\left( {1,2} \right)}$), as well as the twelve- (six-) fold rotation symmetry for $Q_1^{\left( {2,1} \right)}$ ($Q_2^{\left( {2,1} \right)}$), become clearer as *E*_1_ increases. In addition, the ratios of peak values of $I_2^{{\mathrm{HH}}}/I_1^{{\mathrm{HH}}}$ for (1,2) and $I_1^{{\mathrm{HH}}}/I_2^{{\mathrm{HH}}}$ for (2,1) observed in Fig. 3c–f, i.e., 4.1 × 10^−2^ and 1.5 × 10^−2^, are almost respectively reproduced by the calculated intensity ratios of $| {Q_{2}^{( {1,2} )}/Q_{1}^{( {1,2})}}|^{2}$ and $| {Q_1^{( {2,1})}/Q_2^{( {2,1})}} |^{2}$ at *E*_1_ = 10 MV/cm in Fig. 4e, h, i.e., 2.4 × 10^−2^ and 1.1 × 10^−2^. These results evidence that the angular and polarization selection rules of the frequency-mixing process in HHG are modified by the 2D motion of the electron in the non-perturbative interaction regime.

Figure 4i, j illustrates the electron dynamics with help of the three-dimensional plot of the conduction band shown in Fig. 4a. When electrons are driven in the Γ–K direction (sixfold symmetry) by a single linearly polarized laser field **E**_1_ as indicated by the purple broken arrow in the left panel of Fig. 4i, the moving electrons always feel the inversion symmetry in the direction orthogonal to **E**_1_ (the same schematic also applies to the case of two-color parallel polarized light excitation) (Supplementary Fig. 3 in the Supplementary Information IV). Thus, the current in real space can be only induced along the polarization direction of the electric field (Fig. 4i; purple solid arrow in the right panel). On the other hand, adding a small electric-field **E**_2_ that is orthogonal to **E**_1_ modifies the trajectory (Fig. 4j; purple broken arrow in the left panel), which breaks the inversion symmetry along the **E**_2_ direction. As a result, the new trajectory can cause a non-zero group velocity in the **E**_2_ direction, leading to a finite orthogonal current in real space (purple solid arrow in the right panel of Fig. 4j). In addition, because of the inversion symmetry with respect to the Γ point along both Γ–K and Γ–M directions (12-fold symmetry), exactly those (odd,even)- and (even,odd)-order components vanish where an even-order electric field is applied via either the parallel current (i.e., $E_2^{{\mathrm{HH}}}$ = 0 for odd–even combinations) or the orthogonal current (i.e., $E_1^{{\mathrm{HH}}}$ = 0 for even–odd combinations).

Indeed, for **E**_1_ along the Γ–K direction, as shown in Fig. 3c–h, only $I_1^{{\mathrm{HH}}}$ with (1,2)-order as well as only $I_2^{{\mathrm{HH}}}$ with (2,1)- and (4,1)-order are observed at *φ* = 60° × *p*. The same selection rule can apply for **E**_1_ along the Γ–M direction (*φ* = 30° + multiples of 60°), the band curvatures are different between the Γ–K and Γ–M directions, and thus the six-fold rotational symmetry can be seen in Fig. 3c for the $I_1^{{\mathrm{HH}}}$ of order (1,2), and Fig. 3f, h for the $I_2^{{\mathrm{HH}}}$ of orders (2,1) and (4,1). Finally, when **E**_1_ is aligned with the Γ–K or Γ–M directions, the $I_2^{{\mathrm{HH}}}$ of the (1,2) order and the $I_1^{{\mathrm{HH}}}$ of the (2,1) and (4,1) orders vanish. Therefore, the 12-fold rotational symmetries can be seen in Fig. 3d, e, g. These results show that the selection rules of HHs caused by the nonlinear intraband current are determined by the electron trajectory and the order of the harmonics.

**Fig. 5:**
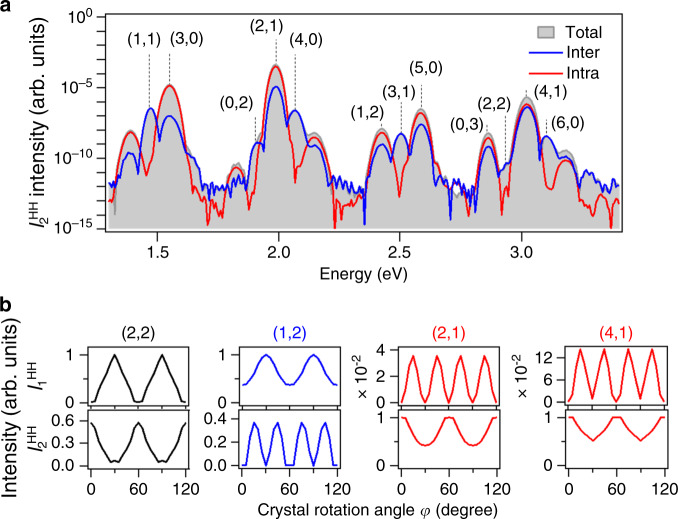
Numerical simulation based on the time-dependent density matrix method. **a** Decomposition of the $I_2^{{\mathrm{HH}}}$ component into interband and intraband contributions. The gray filled region, blue curve, and red curve show the total spectrum, interband, and intraband contributions, respectively, calculated for *φ* = 15°. **b** Calculated *φ* dependence of HH intensities $I_1^{{\mathrm{HH}}}$ and $I_2^{{\mathrm{HH}}}$ for the (2,2), (1,2), (2,1), and (4,1) orders. (Either $I_1^{{\mathrm{HH}}}$ or $I_2^{{\mathrm{HH}}}$ for each order is normalized, and their intensity ratios for the same order are maintained).

To confirm the validity of the analytical calculations, we performed ab-initio calculations. (It is confirmed by the calculation that the timing jitter between two excitation pulses does not change the symmetry of angle dependence of HHs as shown in Supplementary Fig. 4 in the Supplementary Information V.) The shaded areas in Fig. 5a represent the simulated spectra of $I_2^{{\mathrm{HH}}}$ obtained at *φ* = 15°. The blue and red curves show the contributions from the intraband current and the interband current, respectively. In the peaks corresponding to the (2,1) and (1,2) orders, the intraband current is dominant, while the (4,1)-order peak has an interband contribution that is comparable to the intraband contribution. The presence of a significant interband contribution in the (4,1) peak slightly above 3 eV is attributed to the interference of multiple bands^4,9^, because the band edge of the second conduction band is located at 3 eV. Figure 5b shows the calculated *φ* dependence. By incorporating the interband effect, the *φ* dependence of (4,1) and the other orders can be accurately predicted and the symmetry of interband current is discussed with the calculated transition momentum shown in Supplementary Fig. 5 in Supplementary Information VII.

## Discussion

Besides the discussed modification of the selection rule for HHG, the amplification of the nonlinear intraband current obtained by adding the small optical field **E**_2_ has important implications for applications. This effect enables HH emission perpendicular to that generated by the main field **E**_1_, and thus can be used to control the polarization of HHs. For example, at *φ* equal to multiples of 30° and for *E*_1_ = 10 MV/cm and *E*_2_ = 0.5 MV/cm, the $I_2^{{\mathrm{HH}}}$ of the (2,1) order is about 100 times larger than the $I_1^{{\mathrm{HH}}}$ of (2,1) and about 1.5 times larger than the $I_1^{{\mathrm{HH}}}$ of (4,0) [$I_2^{{\mathrm{HH}}}$ of (4,0) vanishes for these angles]. If we employ the condition *ħω*_2_ = 2*ħω*_1_ and **E**_1_ and **E**_2_ are mutually orthogonal, the generated HH with frequency 4*ω*_1_ in the **E**_2_ direction is 1.5 times stronger than that in the **E**_1_ direction, despite the input energy ratio of *E*_1_^2^:*E*_2_^2^ = 400:1 (Supplementary Fig. 6 in Supplementary Information VIII). In this way, the polarization direction of the electric field can be rotated by about 50° with respect to the **E**_1_ direction. Our results clearly showed that the angular and polarization selection rules for frequency-mixing processes are modified by the strongly driven carriers in the regime beyond perturbative nonlinear optics. Since here the response time is only limited by the electron motion, the generation of HHs constitutes an ultrafast polarization control and application to optical switching can be expected.

## Methods

Two orthogonally polarized optical pulses [*ħω*_1_ = 0.517 eV (*λ*_1_ = 2.4 μm) and *ħω*_2_ = 0.954 eV (*λ*_2_ = 1.3 μm)] were generated by separate optical parametric amplifiers with a common Ti:sapphire amplifier (repetition rate: 1 kHz) as a pump source. The sample was a bulk GaSe crystal (*c*-cut) with a thickness of 30 μm. The pulses were focused onto the sample under normal incidence, and the maximum electric-field amplitudes inside the sample were *E*_1_ = 10 MV/cm and *E*_2_ = 0.5 MV/cm. The laser used to apply the field *E*_1_ (10 MV/m) had a pulse width of 100 fs and a spot diameter of ≈60 μm. The pulse energy required to obtain a field strength of 10 MV/cm was 2 μJ. The laser used to apply the orthogonal field *E*_2_ had a pulse duration of 120 fs and a spot diameter of ≈60 μm. To achieve 0.5 MV/cm, we used a pulse energy of 6 nJ. The HHs were detected using a charge-coupled-device camera combined with a spectrometer. In this study, we used a spectrometer (Shamrock 163, Andor Technology Ltd.) equipped with an UV-enhanced CCD (iDus 420 Series, model #DV420A, Andor Technology Ltd.). The crystal-angle dependence of the intensity for different harmonics was measured by rotating the sample with respect to the pulse propagation axis. The emitted HHs had different polarizations, which were analyzed by a polarizer.

## Supplementary information

Supplementary Information

## Data Availability

The data that support the findings of this study are available from the corresponding author upon reasonable request.
