# Supplementary material for: Modifying angular and polarization selection rules of high-order harmonics by controlling electron trajectories in k-space
Source: Nat Commun. 2020 Jun 17;11:3069. doi: 10.1038/s41467-020-16875-5 (PMC7299964; doi:10.1038/s41467-020-16875-5)
Supplement: Supplementary file 1 — Supplementary Information [file 41467_2020_16875_MOESM1_ESM.pdf]

Supplementary Information for

Modifying angular and polarization selection rules of high-order harmonics by controlling electron trajectories in k-space

Yasuyuki Sanari[1], Tomohito Otobe[2], Yoshihiko Kanemitsu[1] & Hideki Hirori[1]

[1]*Institute for Chemical Research, Kyoto University, Uji, Kyoto 611-0011, Japan*

[2]*Kansai Photon Science Institute, National Institutes for Quantum and Radiological Science and Technology (QST), Kizugawa, Kyoto 619-0215, Japan*

I. Crystal angle dependences of generation of odd-order HHs for weak excitation and generation of even-order HHs

The angle dependences of both harmonic orders with $m + n$ = even that originate from interband polarization (Figs. 1**a**–**d**) and orders with $m + n$ = odd for weak excitation (Figs. 1**e**,**f**), can be explained by perturbative nonlinear optics [1]. Figures 1**a**–**d** show the φ dependences of the I_1^{HH} and I_2^{HH} components for (1,1) and (2,2), which is representative of the harmonic orders with $m + n$ = even. Regarding the even order (1,1), I_1^{HH} vanishes at φ = 30 and 90° (Fig. 1**a**), and I_2^{HH} vanishes at φ = 0, 60, and 120° (Fig. 1**b**). To explain the periodicity for $m + n$ = even in terms of conventional nonlinear optics, the nonlinear polarization $P_i^{(m+n)}$ with a nonlinear susceptibility χ can be used. In general, $P_i^{(m+n)}$ reads [2]

$$P_i^{(m+n)} = \chi_{i\underbrace{1\cdots1}_{m}\underbrace{2\cdots2}_{n}} E_1^m E_2^n, (i = 1,2). \quad (1)$$

First, we consider the nonlinear polarization $P_1^{(1+1)} = \chi_{112} E_1^1 E_2^1$ for the (1,1) order at φ = 30 and 90°. As shown in the inset of Fig. 1**a**, $\mathbf{E}_2$ is oriented along the zigzag direction and $\mathbf{E}_1$ and $\mathbf{P}_1$ are oriented along the armchair direction. Thus, the inversion operation that flips the zigzag direction, $\boldsymbol{R_{zig}}$, changes only the sign of $\mathbf{E}_2(t)$, i.e., $\boldsymbol{R_{zig}}[P_1^{(1+1)}] = P_1^{(1+1)}$, $\boldsymbol{R_{zig}}[E_1] = E_1$, and $\boldsymbol{R_{zig}}[E_2] = -E_2$. Since a non-zero χ_{112} would lead to a contradiction, χ_{112} and I_1^{HH} vanish at φ = 30 and 90°. For the same reason, χ_{212} and I_2^{HH} vanish at φ = 0, 60, and 120°. This explanation can be generalized to any mixed harmonic order (m,n) with both m and n being odd. In the case of both m and n being even as shown in Figs. 1**c**,**d**, the angle where χ vanishes can also be determined by considering the inversion operation in the zigzag direction as similarly with (odd, odd) case and is reversed from the case of both m and n being odd. Thus, the six-fold rotation symmetry

of the harmonic orders obeying $m + n$ = even can be explained by the crystal symmetry and conventional nonlinear polarization.

The behaviors of components with $m + n$ = odd observed under weak excitation conditions can be explained by perturbative nonlinear optics. Figures 1**e** and 1**f** show the measured φ dependences of I_1^{HH} and I_2^{HH} for the (1,2) order as a representative for the group of (odd,even) orders. It can be seen that under a weak applied field (E_1 = 5 MV/cm), I_1^{HH} is almost constant and I_2^{HH} is zero. These results can be explained in terms of the $\overline{6}$m2 crystallographic point group of GaSe, which leads to the nonlinear susceptibilities χ_{1122} being constant and χ_{2122} being zero [3]. The angle dependence of the (even,odd) components including (2,1) can be similarly understood in terms of the nonlinear susceptibilities in case of a weak field E_1.

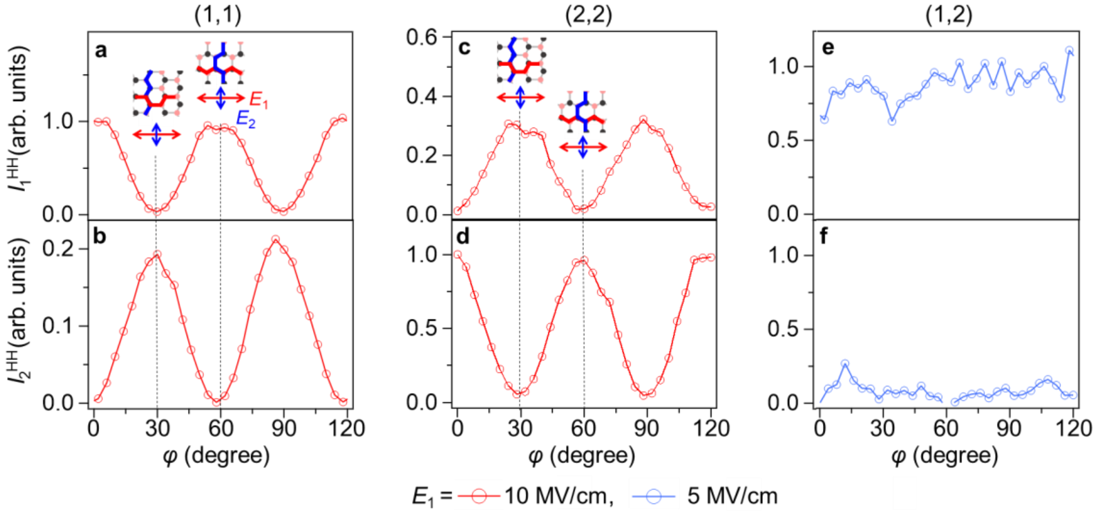

Supplementary Figure 1. The angle dependences of high-order harmonics with *m* + *n* = even and with *m* + *n* = odd. a,**c**, Crystal angle dependences of the I_1^{HH} components for the harmonic orders (1,1) and (2,2), respectively, and **b**,**d**, those of the I_2^{HH} components (E_1=10 MV/cm, E_2=0.5 MV/cm). **e**,**f**, The corresponding dependences in case of weak field E_1 for the (1,2) order (E_1=5 MV/cm, E_2=0.5 MV/cm).

II. Non-perturbative region observed in the intensity dependence

One of the characteristics of HHG is that, above a certain applied field (E_1), the intensity dependence does not follow the scaling law of nonlinear polarization expected from perturbative nonlinear optics. This threshold is often regarded as a characteristic value that separates perturbative and non-perturbative regimes. However, this interpretation is not generally valid. For example, the intensity dependences in Fig. 1**c** (E_1 up to 10 MV/cm) in the main text follow the scaling law. Nevertheless, we observe a non-perturbative behavior in the angle dependence of order (1,2) already at E_1 = 10 MV/cm. Furthermore, the angle dependence does not change near this threshold value. Figure 2**a** shows an extended view of the E_1 dependences in Fig. 1**c** (E_1 up to 16 MV/cm) in the main text. It can be seen that the data follows the scaling law for electric field strengths up to E_1 = 10 MV/cm, but most orders start to deviate from the scaling law above E_1 = 10 MV/cm. The angle dependences of the (1,2) and (2,1) intensities at E_1 = 10 and 15 MV/cm

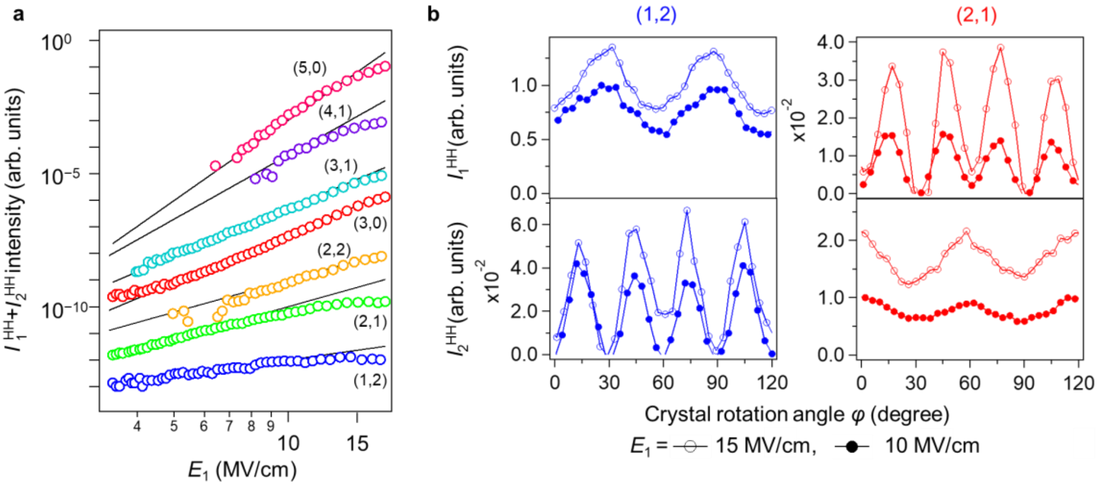

Supplementary Figure 2. Electric-field and crystal-rotation dependences of high-order hamonics for over E_1=10 MV/cm. a, Experimentally measured integrated harmonic intensities $I_1^{\mathrm{HH}}+I_2^{\mathrm{HH}}$ for φ=15° as a function of E_1 up to 16 MV/cm, while E_2=0.5 MV/cm. The black lines are proportional to E_1^{2m} and serve as guides for the eye. The data sets are offset for clarity. **b**, φ dependences of the HH intensity components of the (1,2) and (2,1) orders measured for E_1 = 10 and 15 MV/cm, while E_2=0.5 MV/cm.

are shown in Fig. 2**b**. Although 15 MV/cm is well above the threshold value, the data taken at E_1 = 15 MV/cm (open circles) exhibit the same angle dependences as the non-perturbative angular dependence observed at E_1 = 10 MV/cm (closed circles). This it suggests that the HHG mechanism does not change within this range of electric field strengths. Because a non-perturbative behavior can be observed in the angle dependence even under weaker applied electric fields, it can be said that the angle dependence (Fig. 3 in the main text) is more sensitive to the band anharmonicity than the intensity dependence (Fig. 1**c** in the main text). In addition, we performed the experiment with the higher field of E_2, and confirmed that the sample was destroyed already at $E_2 \approx 3$ MV/cm. Immediately before that, the confirmed angle dependences were similar to those shown in Fig. 3 in the main text.

III. Derivation of analytical formula

To calculate the electric field generated by the 2D electron motion in the band, we start from the semi-classical model [4, 5]. The temporal evolution of the electron wave packet driven by the input fields $\mathbf{E}_1(t)$ and $\mathbf{E}_2(t)$ is described by Bloch's acceleration theorem $dk_i(t)/dt = -(e/\hbar)E_i\cos(\omega_i t), (i = 1,2)$ [6, 7]. In our case we have

$$\mathbf{k}(t) = \big(k_1(t), k_2(t)\big) = \left(-\frac{eE_1}{\hbar\omega_1}\sin(\omega_1 t), -\frac{eE_2}{\hbar\omega_2}\sin(\omega_2 t)\right), \tag{2}$$

where e is the elementary charge, $\hbar$ is the Dirac constant, and the initial state is $k_i(t = 0) = 0$. The group velocity is defined as $\mathbf{v}_\mathrm{g}(\mathbf{k}) = (1/\hbar)(d\epsilon(\mathbf{k})/d\mathbf{k})$ using the energy dispersion $\epsilon(\mathbf{k})$ of the band in which the wave packet moves. The intraband current $\mathbf{J}_\mathrm{intra}(t)$ can be obtained from the product of $\mathbf{v}_\mathrm{g}(\mathbf{k})$ and the electron density distribution $n(\mathbf{k}, t)$, i.e., $\mathbf{J}_\mathrm{intra}(t) = -\int e\mathbf{v}_\mathrm{g}(\mathbf{k})n(\mathbf{k}, t)d\mathbf{k}$. By integrating over the whole Brillouin zone under the assumption of a delta-like electron density $n(\mathbf{k}, t) = \delta(\mathbf{k} - \mathbf{k}(t))$ for the single electron, a simple expression for the intraband current, $\mathbf{J}_\mathrm{intra}(t) = -e\mathbf{v}_\mathrm{g}\big(\mathbf{k}(t)\big)$, can be deduced. The electric field of the HH, $\mathbf{E}^\mathrm{HH}(t)$, which is

generated by the intraband current, can be derived from the time derivative of $\mathbf{J}_{\mathrm{intra}}(t)$:

$$\mathbf{E}^{\mathrm{HH}}(t) \propto \frac{d\mathbf{J}_{\mathrm{intra}}(t)}{dt} = -\frac{e}{\hbar}\frac{d}{dt}\frac{d\epsilon(\mathbf{k})}{d\mathbf{k}} = -\frac{e}{\hbar}\frac{d^2\epsilon(\mathbf{k})}{d\mathbf{k}^2}\frac{d\mathbf{k}(t)}{dt}$$
$$= -\frac{e}{\hbar}\frac{d^2\epsilon(\mathbf{k})}{d\mathbf{k}^2}[\mathbf{E}_1(t) + \mathbf{E}_2(t)]. \quad (3)$$

The component of $d^2\epsilon(\mathbf{k})/d\mathbf{k}^2$ is a rank-two tensor representing the curvature of the band, and Eq. (3) can be expressed using each tensor component as shown below.

$$\begin{pmatrix} E_1^{\mathrm{HH}}(t) \\ E_2^{\mathrm{HH}}(t) \end{pmatrix} \propto \begin{pmatrix} \frac{\partial^2\epsilon(\mathbf{k})}{\partial k_1^2} & \frac{\partial^2\epsilon(\mathbf{k})}{\partial k_1 \partial k_2} \\ \frac{\partial^2\epsilon(\mathbf{k})}{\partial k_2 \partial k_1} & \frac{\partial^2\epsilon(\mathbf{k})}{\partial k_2^2} \end{pmatrix} \begin{pmatrix} E_1\cos(\omega_1 t) \\ E_2\cos(\omega_2 t) \end{pmatrix}, \quad (4)$$

In order to calculate this curvature tensor analytically, the band dispersion of the first conduction band of GaSe was modeled in the k_x–k_y plane using a cosine function:

$$\epsilon(\mathbf{k}) = \epsilon(k_x, k_y) = E_g + b\left[1 - \sum_{j=1,2,3}\cos(a\mathbf{r}_j \cdot \mathbf{k})\right], \quad (5)$$

$$\mathbf{r}_j = \left(\cos\left(\tfrac{2j-1}{3}\pi\right), \sin\left(\tfrac{2j-1}{3}\pi\right)\right), \quad (6)$$

By fitting the band structure calculated by DFT to this cosine function, we obtained E_g = 1.9 eV, a = 13 Å, and b = 0.5 eV. When $\mathbf{E}_1(t)$ and $\mathbf{E}_2(t)$ are applied in the directions that are tilted by the rotation angle φ with respect to the k_x and k_y axes, respectively, the relationship between k_x, k_y and k_1, k_2 becomes $(k_x, k_y) = (k_1\cos\varphi - k_2\sin\varphi, k_1\sin\varphi + k_2\cos\varphi)$. By substituting Eq. (5) into Eq. (4) with this coordinate transformation, the band curvature tensor can be expressed in terms of the cosine band parameters a, b, the position of the electron wave packet in $\boldsymbol{k}$-space (k_1, k_2), and the rotation angle φ:

$$\frac{\partial^2\epsilon(\mathbf{k})}{\partial k_1^2}=\frac{\mathrm{ba}^2}{3}\sum_{j=1,2,3}\cos^2\left(\varphi-\frac{2j-1}{3}\pi\right)\cos\left[a\cos\left(\varphi-\frac{2j-1}{3}\pi\right)k_1(t)-a\sin\left(\varphi-\frac{2j-1}{3}\pi\right)k_2(t)\right], \tag{7}$$

$$\frac{\partial^2\epsilon(\mathbf{k})}{\partial k_1\partial k_2}=\frac{\partial^2\epsilon(\mathbf{k})}{\partial k_2\partial k_1}$$

$$=-\frac{\mathrm{ba}^2}{6}\sum_{j=1,2,3}\sin\left[2\left(\varphi-\frac{2j-1}{3}\pi\right)\right]\cos\left[acos\left(\varphi-\frac{2j-1}{3}\pi\right)k_1(t)-asin\left(\varphi-\frac{2j-1}{3}\pi\right)k_2(t)\right], \tag{8}$$

$$\frac{\partial^2\epsilon(\mathbf{k})}{\partial k_2^2}=\frac{\mathrm{ba}^2}{3}\sum_{j=1,2,3}\sin^2\left(\varphi-\frac{2j-1}{3}\pi\right)\cos\left[a\cos\left(\varphi-\frac{2j-1}{3}\pi\right)k_1(t)-a\sin\left(\varphi-\frac{2j-1}{3}\pi\right)k_2(t)\right]. \tag{9}$$

It can be seen that all three equations contain the same expression for the cosine function with the square brackets. By substituting Eq. (2) into this expression and expanding it in a series of Bessel functions of the first kind, we can rewrite this common expression as follows [7]:

$$\cos\left[a\cos\left(\varphi-\frac{2j-1}{3}\pi\right)\left(-\frac{eE_1}{\hbar\omega_1}\right)\sin(\omega_1 t)-a\sin\left(\varphi-\frac{2j-1}{3}\pi\right)\left(-\frac{eE_2}{\hbar\omega_2}\right)\sin(\omega_2 t)\right]$$

$$=\sum_{M,N=0}^{\infty}\{\left(2+\delta_{M,0}\right)\left(2+\delta_{N,0}\right)J_{2M}\left(A_j\right)J_{2N}\left(B_j\right)\cos(2M\omega_1 t)\cos(2N\omega_2 t)$$
$$+4J_{2M+1}\left(A_j\right)J_{2N+1}\left(B_j\right)\cos[(2M+1)\omega_1 t]\cos[(2N+1)\omega_2 t]\}, \tag{10}$$

$$\mathrm{A_j}=\frac{\omega_{B1}}{\omega_1}\cos\left(\varphi-\frac{2j-1}{3}\pi\right),\mathrm{B_j}=\frac{\omega_{B2}}{\omega_2}\sin\left(\varphi-\frac{2j-1}{3}\pi\right), \tag{11}$$

Here, $\omega_{Bi}=aeE_i/\hbar$ defines the Bloch frequency, and $\delta_{\mathrm{i,j}}$ is the Kronecker delta. Eq. (10) describes a sum of discrete oscillation terms and contains only terms for which the sum of the orders of the two frequency components $[(2M+1)\pm(2N+1)]$ is even. Using

this expansion and Eqs. (4) and (7–9), the generated harmonic electric field can be expanded as follows.

$$E_1^{HH} = \frac{\partial^2 \epsilon(\mathbf{k})}{\partial k_1^2} E_1 \cos(\omega_1 t) + \frac{\partial^2 \epsilon(\mathbf{k})}{\partial k_2 \partial k_1} E_2 \cos(\omega_2 t)$$
$$= \sum_{M,N=0}^{\infty} \{Q_1^{(2M,\pm(2N+1))}(E_1, E_2, \varphi) \cos[2M\omega_1 t \pm (2N+1)\omega_2 t]$$
$$+ Q_1^{(2M+1,\pm 2N)}(E_1, E_2, \varphi) \cos[(2M+1)\omega_1 t \pm 2N\omega_2 t]\}, \qquad (12)$$

$$E_2^{HH} = \frac{\partial^2 \epsilon(\mathbf{k})}{\partial k_1 \partial k_2} E_1 \cos(\omega_1 t) + \frac{\partial^2 \epsilon(\mathbf{k})}{\partial k_2^2} E_2 \cos(\omega_2 t)$$
$$= \sum_{M,N=0}^{\infty} \{Q_2^{(2M,\pm(2N+1))}(E_1, E_2, \varphi) \cos[2M\omega_1 t \pm (2N+1)\omega_2 t]$$
$$+ Q_2^{(2M+1,\pm 2N)}(E_1, E_2, \varphi) \cos[(2M+1)\omega_1 t \pm 2N\omega_2 t]\}, \qquad (13)$$

$$Q_1^{(2M,\pm 2N+1)} = \sum_{j=1,2,3} \left\{ \frac{a^2 b E_1}{3} \cos^2\left(\varphi - \frac{2j-1}{3}\pi\right) \left[J_{2M+1}(A_j) J_{2N+1}(B_j) + (1-\delta_{M,0}) J_{2M-1}(A_j) J_{2N+1}(B_j) \right] - \frac{a^2 b E_2}{24} \sin\left[2\left(\varphi - \frac{2j-1}{3}\pi\right)\right] \left[(1+\delta_{N,0})(2+\delta_{M,0})(2+\delta_{N,0}) J_{2M}(A_j) J_{2N}(B_j) + 2(2+\delta_{M,0}) J_{2M}(A_j) J_{2N+2}(B_j) \right] \right\}, \qquad (14)$$

$$Q_1^{(2M+1,\pm 2N)} = \sum_{j=1,2,3} \left\{ \frac{a^2 b E_1}{12} \cos^2\left(\varphi - \frac{2j-1}{3}\pi\right) \left[(1+\delta_{M,0})(2+\delta_{M,0})(2+\delta_{N,0}) J_{2M}(A_j) J_{2N}(B_j) + 2(2+\delta_{N,0}) J_{2M+2}(A_j) J_{2N}(B_j) \right] - \frac{a^2 b E_2}{6} \sin\left[2\left(\varphi - \frac{2j-1}{3}\pi\right)\right] \left[J_{2M+1}(A_j) J_{2N+1}(B_j) + (1-\delta_{N,0}) J_{2M+1}(A_j) J_{2N-1}(B_j) \right] \right\}, \qquad (15)$$

$$Q_2^{(2M,\pm 2N+1)} = \sum_{j=1,2,3}\left\{-\frac{a^2bE_1}{6}\sin\left[2\left(\varphi-\frac{2j-1}{3}\pi\right)\right]\left[J_{2M+1}(A_j)J_{2N+1}(B_j) + (1-\delta_{M,0})J_{2M-1}(A_j)J_{2N+1}(B_j)\right] + \frac{a^2bE_2}{12}\sin^2\left(\varphi-\frac{2j-1}{3}\pi\right)\left[(1+\delta_{N,0})(2+\delta_{M,0})(2+\delta_{N,0})J_{2M}(A_j)J_{2N}(B_j) + 2(2+\delta_{M,0})J_{2M}(A_j)J_{2N+2}(B_j)\right]\right\}, \quad (16)$$

$$Q_2^{(2M+1,\pm 2N)} = \sum_{j=1,2,3}\left\{-\frac{a^2bE_1}{24}\sin\left[2\left(\varphi-\frac{2j-1}{3}\pi\right)\right]\left[(1+\delta_{M,0})(2+\delta_{M,0})(2+\delta_{N,0})J_{2M}(A_j)J_{2N}(B_j) + 2(2+\delta_{N,0})J_{2M+2}(A_j)J_{2N}(B_j)\right] + \frac{a^2bE_2}{3}\sin^2\left(\varphi-\frac{2j-1}{3}\pi\right)\left[J_{2M+1}(A_j)J_{2N+1}(B_j) + (1-\delta_{N,0})J_{2M+1}(A_j)J_{2N-1}(B_j)\right]\right\}, \quad (17)$$

Here, the coefficients $Q_{\mathrm{i}}^{(m,\pm n)}$ in front of the $\cos[(m\omega_1 \pm n\omega_2)t]$ terms in $\mathbf{E}_{\mathrm{i}}^{\mathrm{HH}}(t)$ [Eqs. (12) and (13)] are functions of E_1, E_2, and φ. $Q_{\mathrm{i}}^{(m,\pm n)}$ describes how the electric field amplitude of each harmonic order depends on the rotation angle and applied field strength. The parity of the Bessel functions in Eqs. (14–17) does not change by the difference of (M, N), and thus the selection rule resulting in the 12-fold and 6-fold rotational symmetries is the same for higher orders.

IV. HHG using two parallel polarized laser fields

Figure 3**a** shows the crystal rotation angle dependences obtained using two-color laser fields with parallel polarization (I_1^{HH} is shown on the left, and I_2^{HH} on the right side). The employed maximum electric field amplitudes inside the sample are E_1 = 10 MV/cm and E_2 = 0.5 MV/cm. All harmonic orders that satisfy $m + n$ = odd have a six-fold rotation symmetry for I_1^{HH} and a twelve-fold rotation symmetry for I_2^{HH}. These angle and polarization dependences are same as those obtained under single-color excitation, e.g. the same dependence as that of (5,0). In order to calculate these results analytically as in the case of orthogonal polarization, the term $E_1\cos(\omega_1 t) + E_2\cos(\omega_2 t)$ is substituted for the term $E_1\cos(\omega_1 t)$ and the value zero is substituted for the term $E_2\cos(\omega_2 t)$ in Eq. (4). The left and right panels of Figure 3**b** plot the 2D maps of the prefactors that

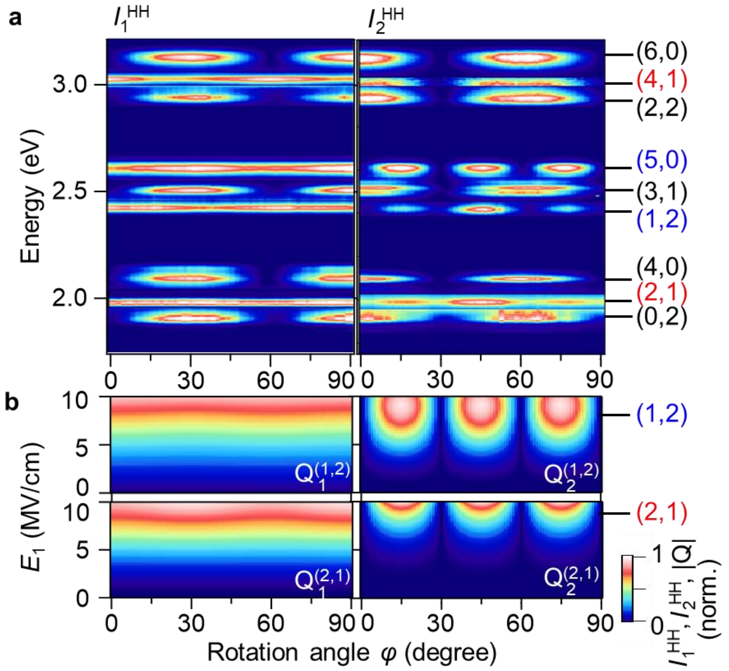

Supplementary Figure 3. Crystal angle dependences of high-order harmonics with parallel electric fields E₁ and E₂. a, Crystal rotation angle dependence of the I_1^{HH} and I_2^{HH} components of the HHG spectrum obtained using two-color laser fields with parallel polarization (E_1=10 MV/cm, E_2=0.5 MV/cm), which are experimentally obtained. **b**, Calculated angle dependence of the prefactors Q for the harmonic orders (1,2) and (2,1). Each plot is normalized.

reflect the electric field amplitude in Eqs. (14–17), that is, Q, for the (1,2) and (2,1) orders, respectively. The theoretical results accurately reproduce the experiment. Similar to the case of single linearly polarized excitation, we find that, when electrons oscillate along a one-dimensional trajectory, the sum of the orders m and n determines the angle dependence.

V. Time-dependent density matrix calculation

We solve the time-dependent equation of motion using the one-body density-matrix representation; the basic equation in the velocity gauge is written as

$$i\hbar \frac{d}{dt}\boldsymbol{\rho}(t) = [h(t), \boldsymbol{\rho}(t)],$$
$$h(t) = h_0 + \frac{e}{mc}\hat{\mathbf{p}} \cdot \mathbf{A}(t) + \frac{e^2}{2mc^2}|\mathbf{A}(t)|^2 + e^{-i\frac{e}{c}\mathbf{A}(t)\cdot\mathbf{r}}\Sigma_0 e^{+i\frac{e}{c}\mathbf{A}(t)\cdot\mathbf{r}}, \quad (18)$$

where $\boldsymbol{\rho}$, h_0, m, c, and $\hat{\mathbf{p}}$ are the density matrix, field-free Hamiltonian, electron mass, light speed, and momentum operator, respectively. The vector potential, $\mathbf{A}(t)$, is determined from the applied electric field (details are explained further below). The initial density matrix $\boldsymbol{\rho}(t = 0)$ is the ground state of h_0. Density-functional theory within the local density approximation (LDA) is employed to calculate h_0. Since the LDA underestimates the band-gap energies of semiconductors, we reproduce the direct band gap of GaSe (1.98 eV) by using the scissors operator Σ_0. Phase factors are introduced on both sides of the scissors operator, since this is a nonlocal operator. The details of this computational approach can be found in previous papers [4]. It should be noted that Eq. (18) does not include many-body effects.

For $\boldsymbol{\rho}(t)$, we select a proper basis set $\{\varphi_{i,\mathbf{k}}\}$, $\rho_{ij}^{\mathbf{k}}(t) = \langle\varphi_{i,\mathbf{k}}|\hat{\boldsymbol{\rho}}(t)|\varphi_{j,\mathbf{k}}\rangle$, where i and $\mathbf{k}$ are the band index and Bloch wave vectors, respectively. The electronic current density $\mathbf{J}_{\mathrm{tot}}(t)$ is calculated using

$$\mathbf{J}_{\mathrm{tot}}(t) = -\frac{1}{\Omega}\sum_{i,\mathbf{k}} e\left\langle \psi_{i,\mathbf{k}}(t)\middle|\hat{\mathbf{p}} + \frac{e}{c}\mathbf{A}(t)\middle|\psi_{i,\mathbf{k}}(t)\right\rangle$$

$$= \sum_{ij,\mathbf{k}} \rho_{ij}^{\mathbf{k}}(t)\left(\mathbf{p}_{ji}^{\mathbf{k}}(t) + \delta_{ij}\frac{e}{c}\mathbf{A}(t)\right) \quad (19)$$

where $\psi_{i,\mathbf{k}}(t)$ is the time-dependent wave function of a certain band i for the wave vector $\mathbf{k}$, $\mathbf{p}_{ji}^{\mathbf{k}}(t)$ is the transition momentum defined as $\mathbf{p}_{ji}^{\mathbf{k}}(t) = \langle\varphi_{j,\mathbf{k}}|\hat{\mathbf{p}}|\varphi_{i,\mathbf{k}}\rangle$, δ_{ij} is the Kronecker delta, and Ω is the volume of the unit cell.

The choice of the basis set is crucial for accurate calculation of the interband and intraband currents. Here, we employ the instantaneous eigenfunctions of $h(t)$, $h(t)\tilde{\varphi}_{i,\mathbf{k}} = \tilde{\epsilon}_{i,\mathbf{k}}\tilde{\varphi}_{i,\mathbf{k}}$. We assume that $h(t)$ does not include many-body effects, which implies that the time-dependence of $h(t)$ depends only on the time-dependent potential $\mathbf{A}(t)$. Then, the one-body time-dependent Schrödinger equation (TDSE) reads

$$i\hbar\frac{\partial u_{i,\mathbf{k}}(t)}{\partial t} = h_{\mathbf{k}}(t)u_{i,\mathbf{k}}(t), \quad (20)$$

$$h_{\mathbf{k}}(t) = \left[-\frac{1}{2m}\left(\hat{\mathbf{p}} + \mathbf{k} + \frac{e}{c}\mathbf{A}(t)\right)^2 + V(\mathbf{r}) + e^{-i\frac{e}{c}\mathbf{A}(t)\cdot\mathbf{r}}\Sigma_{\mathbf{k}}(t)e^{i\frac{e}{c}\mathbf{A}(t)\cdot\mathbf{r}}\right]. \quad (21)$$

Here, $V(\mathbf{r})$ is the potential including the mean field derived by DFT and the Coulomb potentials from the atoms, and $u_{i,\mathbf{k}}(t)$ is the spatially periodic part of $\psi_{i,\mathbf{k}}(t)$, $\psi_{i,\mathbf{k}}(t) = e^{i\mathbf{k}\cdot\mathbf{r}}u_{i,\mathbf{k}}(t)$. The $\mathbf{k}$-dependent Hamiltonian, $h_{\mathbf{k}}(t)$, corresponds to that with the shifted Bloch wave vector $\mathbf{k} \rightarrow \mathbf{k} + e/c\mathbf{A}(t)$. Therefore, $\tilde{\varphi}_{i,\mathbf{k}}$ is the eigenstate of the h_0 with $\mathbf{k} + e/c\mathbf{A}(t)$, and if the electron dynamics are adiabatic, $\tilde{\varphi}_{i,\mathbf{k}}$ is a reasonable approximation for $\psi_{i,\mathbf{k}}(t)$.

The intraband and interband currents are evaluated using

$$\mathbf{J}_{\text{intra}}(t) = -\frac{1}{\Omega}\sum_{i,\mathbf{k}} e\Delta n_{i,\mathbf{k}}(t)\left\langle \tilde{\varphi}_{i,\mathbf{k}} \middle| \hat{\mathbf{p}} + \frac{e}{c}\mathbf{A}(t) \middle| \tilde{\varphi}_{i,\mathbf{k}} \right\rangle$$
$$= \sum_{i,\mathbf{k}} \tilde{\rho}_{ii}^{\mathbf{k}}(t)(\tilde{\mathbf{p}}_{ii}^{\mathbf{k}}(t) + \frac{e}{c}\mathbf{A}(t)) \qquad (22)$$

and

$$\mathbf{J}_{\text{inter}}(t) = \sum_{ij(i\neq j),\mathbf{k}} \tilde{\rho}_{ij}^{\mathbf{k}}(t)\tilde{\mathbf{p}}_{ji}^{\mathbf{k}}(t), \qquad (23)$$

respectively. Here $\Delta n_{i,\mathbf{k}}(t) = n_{i,\mathbf{k}}(t) - n_{i,\mathbf{k}}(t = 0)$ is the transient population change of the state $\tilde{\varphi}_{i,\mathbf{k}}$, and $\tilde{\rho}_{ij}^{\mathbf{k}}$ and $\tilde{\mathbf{p}}_{ji}^{\mathbf{k}}$ are the density matrix and the transition momentum with respect to the eigenfunction $\tilde{\varphi}_{i,\mathbf{k}}$.

Since we applied two orthogonally polarized fields $\mathbf{E}_1(t) = E_1(t)\cos(\omega_1 t)\,\mathbf{e}_1$ and $\mathbf{E}_2(t) = E_2(t)\cos(\omega_2 t)\,\mathbf{e}_2$, the calculation uses a vector potential of the form $\mathbf{A}(t) = \mathbf{A}_1 f(t)\sin[\omega_1(t - 1/2\,\tau)] + \mathbf{A}_2 f(t)\sin[\omega_2(t - 1/2\,\tau)]$ for $0 \le t \le \tau$, and $\mathbf{A}(t) = 0$ otherwise. The vector potential and electric field are connected by the relation, $\mathbf{A}(t) = -c\int_0^t \boldsymbol{E}(t')dt'$. We used the pulse envelope function of $f(t) = \cos^4(\pi t/\tau - \pi/2)$, which is a good approximation of a Gaussian shape and thus provides a sharper spectrum while reducing computational costs. The electric field intensities are E_1 = 10 MV/cm and E_2 = 1 MV/cm, the photon energies are $\hbar\omega_1 = 0.517$ eV and $\hbar\omega_2 = 0.954$ eV, and the pulse duration parameter is τ = 383 fs, which corresponds to a temporal full width at half-maximum of 100 fs. We used 38 valence bands and 65 conduction bands. In order to obtain the angular dependence of the HH components, 64×64×12 points in the first Brillouin zone had to be sampled. We used a time step of $\Delta t =$ 1.0 atomic units (= 24.19 as). The number of time steps is 20000 steps, which corresponds to 483.8 fs. To obtain clear discrete peaks in the spectrum above the band gap, we introduced a relaxation time of 4.03 fs [8]. It should be noted that the HHG spectrum of the total current includes the cross term from interband and intraband currents, $2Re\ [\tilde{J}_{\text{inter}}^{*}(\omega)\ \tilde{J}_{\text{intra}}(\omega)]$.

VI. Calculation for the other orders and timing jitter

In this section, we briefly discuss the possible effects of coherence/timing jitter between the two excitation pulses described by $\mathbf{E}_1(t)$ and $\mathbf{E}_2(t)$. In our experiment, the about 12 cycles of $\mathbf{E}_1(t)$ and 23 cycles of $\mathbf{E}_2(t)$ are included within the pulse duration of 100 fs. Our laser pulses have random time delays (a small timing jitter is present between the two pulses due to, for example, thermal drift, although a common pump laser was employed) and thus the experimental results are the values averaged over the timing jitter. To confirm that this timing jitter does not significantly affect our reported results for the angular dependence, we calculated the angular dependences for different phase offsets as shown in Fig. 4 (phase delay 0 is the timing of the field peaks of the two pulses overlap**)**. The results show that the symmetry of the angular dependence does not change for a different timing between the pulses and we were able reproduce the behaviors of the experimentally observed angular dependences. This data reproduces the experimentally observed angular selection rules. We can interpret this important result as follows: because the optical frequencies of the two pulses are incommensurate, even if the phase of one pulse shifts, the peak-to-peak situation occurs at another timing (within the 100 fs).

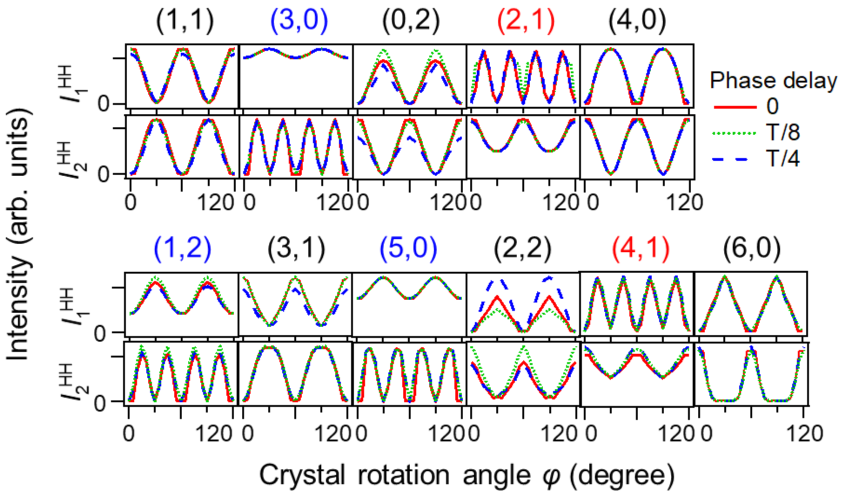

Supplementary Figure 4. Angle-dependences of high-order harmonics for different phase delays. Calculated angular dependences for different phase offsets: 0, $T/8$, and $T/4$. The time period T is that for $\mathbf{E}_2$ pulse.

VII. Symmetry of angle dependences of interband current

Qualitative considerations for the symmetry of the angle dependences of the interband current can be added to the discussion. The interband current can be written as $\mathbf{J}_{\mathrm{inter}}(t) \propto \sum_{k}(d/dt)[\mathbf{d}(\mathbf{k})s(\mathbf{k},t)]$ by using the transition dipole moment $\mathbf{d}(\mathbf{k})$ and the interband polarization $s(\mathbf{k}, t)$. Figure 5 shows a two-dimensional map of the transition momentum $\mathbf{p}(\mathbf{k})$ between the first conduction band and the valence band. The $\mathbf{p}(\mathbf{k})$ and $\mathbf{d}(\mathbf{k})$ are connected by the well-known relation, $m(\varepsilon^h(\mathbf{k}) - \varepsilon^e(\mathbf{k}))\mathbf{d}(\mathbf{k}) = i\hbar\mathbf{p}(\mathbf{k})$. Note that $\mathbf{p}(\mathbf{k})$ has an inversion symmetry with $\varphi = 30 \times n°$ like the first conduction band we modeled in Sec. III. The symmetry of $s(\mathbf{k}, t)$ follows this characteristic, because both $\mathbf{p}(\mathbf{k})$ and the band dispersion $\varepsilon(\mathbf{k})$ have the same symmetry, and time evolution is described by the following expression [7].

$$i\hbar\frac{\partial s(\mathbf{k},t)}{\partial t} = \left(\varepsilon^e(\mathbf{k}) + \varepsilon^h(\mathbf{k}) - \mathrm{i}\frac{\hbar}{T_2}\right)s(\mathbf{k},t) - \left(1 - n^e(\mathbf{k},t) - n^h(\mathbf{k},t)\right)\mathbf{d}(\mathbf{k})\mathbf{E}(t) + ie\mathbf{E}(t)\cdot\nabla_{\boldsymbol{k}}s(\mathbf{k},t), \qquad (24)$$

$$\hbar\frac{\partial n^{e(h)}(\mathbf{k},t)}{\partial t} = -2Im[\mathbf{d}(\mathbf{k})\mathbf{E}(t)s^*(\mathbf{k},t)] + e\mathbf{E}(t)\cdot\nabla_{\mathbf{k}}n^{e(h)}(\mathbf{k},t), \qquad (25)$$

Thus, considering the time-evolution of the wave packet $\mathbf{k}(t)$ for electron-hole pair similarly with the case of intraband current, intraband and interband current have the same

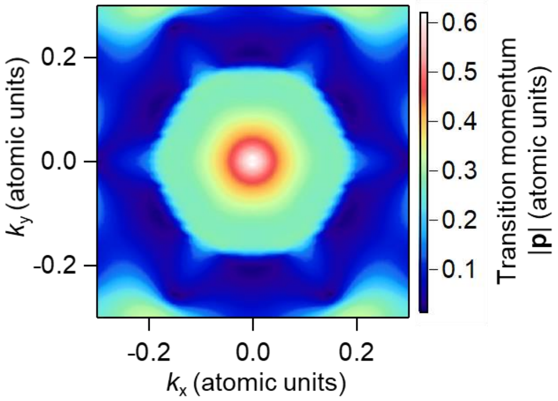

Supplementary Figure 5. Calculation of transition momentum $|\mathbf{p}|$. The transition momentum $|\mathbf{p}|$ between the bottom of the conduction band and top of the valence band is calculated in **k**-space.

symmetry with respect to $\mathbf{k}(t)$. Therefore, the selection rule resulting in the 12-fold and 6-fold rotational symmetries for orders like (4,1) for the interband current is the same as that for the intraband current.

VIII. Efficiency of conversion via the orthogonal current

Figure 6 provides a comparison of I_1^{HH} (red line) and I_2^{HH} (blue line) spectra obtained for φ = 30° as shown in Figs. 2**a** and 2**b** in the main text. Here, the energy ratio of E_1 = 10 MV/cm to E_2 = 0.5 MV/cm is 400:1. Note that the peaks of (4,0), (5,0), and (6,0) (that is, HHs that only include the frequency of $\mathbf{E}_1$) are generated only in the component I_1^{HH}, and the sum frequency peaks of (2,1), (3,1) and (4,1), which are at energies close to those of the (4,0), (5,0), and (6,0) orders, respectively, are generated only in the component I_2^{HH}. In particular, the peak intensity of (2,1), which is the strongest order in the $\mathbf{E}_2$ direction, is 1.5 times stronger than that of (4,0). This means that, if the frequency of $\mathbf{E}_2$ is twice the frequency of $\mathbf{E}_1$ and both fields are mutually orthogonal, the HH generated at frequency $4\omega_1$ in the $\mathbf{E}_2$ direction can be stronger than that in the $\mathbf{E}_1$ direction, despite the input energy ratio of 400:1. This visualizes the high conversion efficiency obtained under excitation with the additional orthogonally polarized electric field. Since the phase of the generated HH can be changed by controlling the phase of the

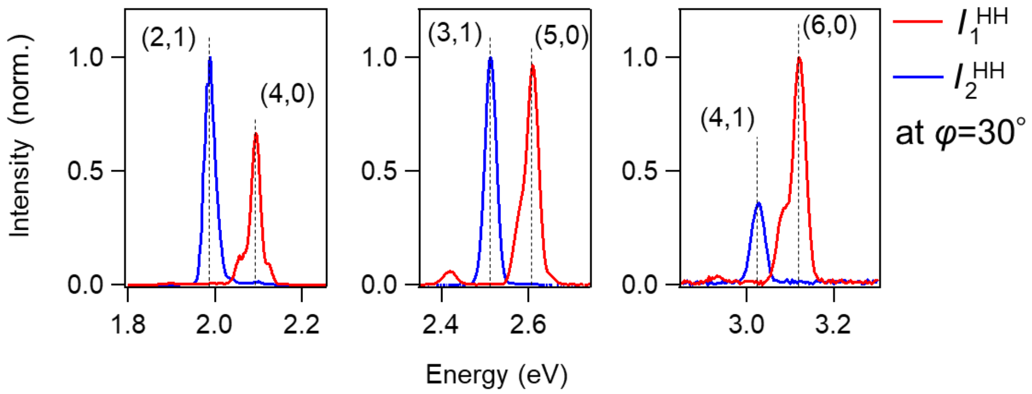

Supplementary Figure 6. The comparison between I_1^{HH} and I_2^{HH}. The I_1^{HH} (red line) and I_2^{HH} (blue line) spectra obtained for φ =30° (E_1=10 MV/cm, E_2=0.5 MV/cm). The intensities are normalized in each region.

input pulse, it can be converted into linearly polarized light by choosing an appropriate phase delay, and the polarization direction of the electric field can be rotated by about 50° with respect to the $\mathbf{E}_1$ direction. This constitutes one approach to realize polarization control using the HHG process.

Supplementary References

[1] Boyd, R. W. Nonlinear Optics, 3rd ed. (Academic Press, San Diego, 2008).

[2] Zavelishko, V. I., Martynov, V. A., Saltiel, S. M., & Tunkin, V. G. Optical nonlinear fourth- and fifth-order susceptibilities. *Sov. J. Quantum Electron.* **5**, 1392 (1975).

[3] Banks, P. S., Feit, M. D., & Perry, M. D. High-intensity third-harmonic generation. *J. Opt. Soc. Am. B* **19**, 102 (2002).

[4] Kaneshima, K., Shinohara, Y., Takeuchi, K., Ishii, N., Imasaka, K., Kaji, T., Ashihara, S., Ishikawa, K. L., & Itatani, J. Polarization-resolved study of high harmonics from bulk semiconductors. *Phys. Rev. Lett.* **120**, 243903 (2018).

[5] Schubert, O., Hohenleutner, M., Langer, F., Urbanek, B., Lange, C., Huttner, U., Golde, D., Meier, T., Kira, M., Koch, S. W., & Huber, R. Sub-cycle control of terahertz high-harmonic generation by dynamical Bloch oscillations. *Nat. Photon.* **8**, 119 (2014).

[6] Krieger, J. B., & Iafrate, G. J. Time evolution of Bloch electrons in a homogeneous electric field. *Phys. Rev. B* **33**, 5494 (1986).

[7] Golde, D., Meier, T., & Koch, S. W. High harmonics generated in semiconductor nanostructures by the coupled dynamics of optical inter- and intraband excitations. *Phys. Rev. B* **77**, 075330 (2008).

[8] Floss, I., Lemell, C., Wachter, G., Smejkal, V., Sato, S. A., Tong, X.-M., Yabana, K., & Burgdorfer, J. *Ab initio* multiscale simulation of high-order harmonic generation in solids. *Phys. Rev. A* **97**, 011401 (R) (2018).
